# Pregnancy, Motherhood and Partner Support in Visually Impaired Women: A Qualitative Study

**DOI:** 10.3390/ijerph19074308

**Published:** 2022-04-04

**Authors:** Elena Commodari, Valentina Lucia La Rosa, Giuseppina Susanna Nania

**Affiliations:** Department of Educational Sciences, University of Catania, 95124 Catania, Italy; e.commodari@unict.it (E.C.); susannanania@virgilio.it (G.S.N.)

**Keywords:** visual impairment, women, pregnancy, motherhood, social support

## Abstract

Background: This qualitative study aimed to explore the experiences of women with vision impairments regarding the meaning of motherhood and their mothering-related issues and priorities. Methods: In-depth individual, semi-structured interviews were conducted between July and December 2020 with a group of visually impaired mothers residing in Italy. The interviews explored experiences related to pregnancy, childbirth, and motherhood; support received from partners, family, and friends; ways of interacting and communicating with the child; and the participants’ sense of personal self-efficacy and self-awareness. Results: Fifteen women participated in this study, ten with a congenital visual impairment and five with an acquired disability. The mean age of the sample was 49 years. The qualitative content analysis of the transcripts of the interviews pointed out four main themes or categories: (1) pregnancy and motherhood experiences, (2) family and social support, (3) relationship and communication with the child, and (4) self-efficacy and self-awareness. Conclusions: This study underlined that mothers with visual impairments show a strong desire to be recognized and accepted as women and mothers by their social environment. Adequate social and family support is associated with a better sense of personal self-efficacy and greater confidence in one’s skills as a mother.

## 1. Introduction

According to the World Health Organization [1], at least 2.2 billion people globally have a vision impairment or blindness. Specifically, 43.3 million people worldwide are blind, and 54% are women [2]. 

Women with disabilities such as vision impairments may experience several aspects of discrimination, including a lack of barrier-free facilities or social and work exclusion [3]. Furthermore, they are often considered incapable of fulfilling traditional gender roles, for example, the maternal role [4]. In this context, the issue of pregnancy and motherhood for disabled women is still underestimated or evaluated critically, and their parenting abilities are often questioned [5,6]. 

According to the literature on the topic, women with physical disabilities face different reactions and attitudes to their pregnancy, including opposition and skepticism [7]. 

In this regard, Schildberger et al. conducted in-depth individual, semi-structured interviews with ten Austrian mothers with various physical or sensory impairments. Participants experienced limited acceptance of their life decisions and discriminatory attitudes. Similarly, another qualitative study by Grue et al. conducted on 30 women aged between 28–49 with different physical disabilities highlighted that these women reported enormous efforts to present themselves and their children as “normal” to be accepted as “ordinary” mothers. This skepticism that mothers with disabilities very frequently experience may be explained by the fact that people with disabilities are still viewed as nonautonomous and dependent on the support of others.

In addition, these mothers may experience different levels of formal and informal support and various types of stressors [8]. The qualitative study by Prilleltensky on mothers’ experiences with physical disabilities underlined the need for these mothers to receive both instrumental and social–emotional support. Specifically, lack of adequate support from informal networks (family, friends, etc.) is associated with intense feelings of frustration and high stress levels. Formal support systems are also critical in facilitating the activities of mothers with disabilities and fostering a positive experience of motherhood.

In this regard, despite the growing number of women with disabilities who wish to be mothers [5,6,9], their desire often clashes with the lack of appropriate specialists who should ensure support and help. Indeed, healthcare specialists tend to be skeptical regarding the effective ability of women with disabilities to conduct a pregnancy and take care of a child. As a result, they seem to pay more attention to diagnoses instead of the functional and maternal abilities of the pregnant and postpartum woman [10,11]. 

Available data on the experience of motherhood in women with visual impairments are derived primarily from qualitative research, such as that of Conley-Jung et al. [12] who interviewed 42 visually impaired mothers. According to this study, the degree of visual impairment does not appear to be significantly associated with the preparation for child care tasks or initial reactions to pregnancy. Furthermore, the main concerns reported by these mothers included the safety of their children, the time needed to adjust to their new status as a mother with disabilities, and dealing with others’ reactions to motherhood [12].

Another qualitative study by Moghadam et al. [13] reported the parenting experiences of blind mothers in Iran, using a hermeneutic phenomenology approach. Specifically, the authors conducted semi-structured, in-depth interviews to produce qualitative data with nine blind mothers. According to the results of this study, blindness changes the mothering behaviors of blind women, and they often adopt a close-mothering approach in caring for their children to cope with their limitations and decrease their level of child-related anxiety [13].

Motherhood and mother–child communication can be undermined when the mother is visually impaired [12,13]. Indeed, sighted mothers primarily understand their children’s needs through visual signs, such as facial expressions. Nevertheless, a mother who is blind can successfully interact with the child using alternative strategies to interpret their child’s signals and respond sensitively [13]. In this regard, it has been reported that blind mothers successfully interact with their children and use multiple strategies to understand and respond to their child’s signals [13]. Confirming this, many research works supported the maternal skills of women with disabilities who wished to be mothers and their ability to develop alternative approaches to contrast the typical difficulties of motherhood [7,14,15,16]. 

In this scenario, understanding the lived experiences of visually impaired mothers can contribute to valuable knowledge in providing appropriate health care and educational services for women who are blind and their children [13].

A review of the current literature showed that research about the mothering experiences of visually impaired women who provide care for their children is still quite limited. Therefore, this qualitative study aimed to investigate the experiences of women with visual impairments regarding the meaning of motherhood and their mothering-related issues and priorities. Specifically, we explored experiences related to pregnancy, childbirth, and motherhood; support received from partners, family, and friends; ways of interacting and communicating with the child; and the sense of personal self-efficacy and self-awareness of the women interviewed. Our choice to use a qualitative methodology is related to the fact that almost all available studies on the topic have been conducted with qualitative approaches, and this allows us to compare our results with data from the literature.

## 2. Materials and Methods

Visually impaired mothers residing in Italy were recruited to participate in this study through the Italian Union of the Blind and Partially Sighted, a private organization that represents and protects the interests and needs of people with visual impairments in Italy. In-depth individual, semi-structured interviews were conducted between July and December 2020. Regarding the inclusion and exclusion criteria, women with congenital or acquired visual impairment before pregnancy were recruited for this study. Mothers with psychiatric comorbidities or mental disabilities were excluded from the sample. Neither the mothers’ ages nor the ages of the children were considered exclusion criteria in the study.

Regarding sample size, according to Hennink and Kaiser [17], 9–17 interviews are generally considered appropriate for adequate data saturation in qualitative studies. Therefore, we considered a sample of 15 women with visual impairment to be adequate.

The research team structured a guide for conducting the interviews by identifying the experience of pregnancy, childbirth, and the relationship with the child as the central themes to be explored. Specifically, the interviews focused on how the women reacted to the news of their pregnancy, the support they received from the partner, the family, and their social environment, how they prepared for childbirth and motherhood, and the prejudices related to women with disabilities becoming mothers that they faced. Furthermore, the women interviewed completed the *General Self-Efficacy Scale* (GSE) to investigate each mother’s belief in their ability to cope with disability and motherhood [18]. The scale consists of 10 items (i.e., “Thanks to my resourcefulness, I can handle unforeseen situations”). All items are answered on a four-point scale ranging from 1 = “not at all true” to 4 = “precisely true”, with a total score between 10 and 40 [18]. A higher score indicated more self-efficacy [19]. In addition, the scale shows good psychometric characteristics of reliability (Cronbach’s α between 0.76 and 0.90).

Due to the COVID-19 pandemic restrictions, interviews were conducted by telephone. In this regard, a small but growing body of literature has documented that telephone in-depth interviews are a useful option for qualitative research and that data quality is comparable between face-to-face and telephone interviews [20]. Miller [21] underlined that telephone interviews allow researchers to reach people who might not otherwise be able to participate in the research and have their views represented. Furthermore, telephone interviews have proven to be more suitable for vulnerable and marginalized populations and more sensitive questions [20,22], as in the case of our study. Therefore, the telephone interviews were conducted following the indications of the literature on the topic [20,21,22] in order not to compromise the quality of the data collected. 

The interviewer was a blind female researcher supported by a sighted researcher in case of any technical problems to facilitate the compliance of the women participating in the study.

After explaining the purpose of the interview and obtaining the informed consent of the women recruited to participate in the research, the telephone interviews were recorded with the participants’ permission and then transcribed, preserving the anonymity of the data. Each interview lasted on average 30 to 50 min, and the duration of the interview was in no way affected by the use of the telephone, which allowed optimal time management as already documented in other studies that have used this tool [20,21,22].

Qualitative data obtained from the interviews were analyzed using the qualitative content analysis proposed by Mayring [23,24], which has already been applied in the qualitative study by Schildberger et al. [3] on the experiences of Austrian mothers with mobility or sensory impairments during pregnancy, childbirth, and the puerperium. 

According to inductive category development [23], qualitative data were coded and analyzed to show emerging themes [25,26]. Each interview transcript was carefully reviewed, the extracted data were classified, and the categories were then grouped into broader themes. The development and confirmation of the thematic coding structure involved two researchers who conducted an individual and recursive reading of the textual data and discussed the emerging themes extracted from the interviews. The researchers examined any discrepancies, reaching a consensus [27]. The agreement on the content analysis was assessed and was high [26]. Then, coding rules were formulated for each category to determine precisely when a particular passage of text can be coded in a specific category (deductive category application) [23]. 

The study was performed following the ethical standards of the Ethical Code for Italian psychologists (L. 18.02.1989, n. 56), Italian law for data privacy (DLGS 196/2003), and the Ethical Code for Psychological Research (27 March 2015) approved by the Italian Psychologists Association. No sensitive data that could identify the participants was collected. The Internal Review Board of the Department of Educational Sciences, University of Catania, approved this study.

## 3. Results

Fifteen women participated in this study, ten with a congenital visual impairment and five with an acquired disability. The mean age of the sample was 49 years. Nine women had one child, while six women had two children. At the time of the interviews, eight women were married, two were cohabitants, four were divorced, and one was a widow. A list of the study participants and their pseudonyms is reported in Table 1.

The qualitative content analysis of the transcripts of the interviews pointed out four main themes or categories: (1) pregnancy and motherhood experiences, (2) family and social support, (3) relationship and communication with the child, and (4) self-efficacy and self-awareness.

### 3.1. Pregnancy and Motherhood Experiences

The women interviewed first described their own reactions to their pregnancy and their partners, family members, and friends.

All participants welcomed the news of their pregnancy with great joy. In particular, the strong desire to have a child is often mentioned in the answers of the interviewed mothers. The fun and happiness of pregnancy are closely linked to the desire for motherhood expressed by the women in our sample:

*M7: “When I found out I was pregnant, I felt great joy and happiness because I had wanted it so much.”*

*M12: “I felt immense joy, I wanted with all my heart to become a mother, and finally, my dream was coming true.”*

*M15: “Obviously beautiful. In the first pregnancy, I was very young, and I experienced the news of the pregnancy differently. When I heard about the second pregnancy, I was super happy because I wanted it so much. For my first daughter, I didn’t expect to get pregnant a month after getting married.”*

The participants also described their emotions during pregnancy and immediately after delivery. When asked how they imagined their child during pregnancy, most said they imagined the child to be beautiful but above all healthy. Indeed, concern about the risk that the child might inherit their visual problems is a recurring theme in the stories of the women interviewed. Two women reported that they preferred not to imagine their baby because they knew they would never see him, but, again, their main concern was that the baby would be healthy.

*M5: “I imagined him to be handsome. I always prayed to the Lord that he would be healthy and beautiful. My son grew up well, very intelligent, handsome.”*

*M7: “Brat definitely, and indeed he is. Physically different from reality. But what mattered most was that he was healthy.”*

*M8: “I didn’t have any expectations as I was already happy to be pregnant, so I tried not to imagine him because I knew I would never be able to see him.”*

*M9: “I preferred not to imagine him but to feel him growing inside me because imagining him made me remember that I would never be able to see him with my own eyes, and it made me sad.”*

When asked if they had ever dreamt of their child during pregnancy, most said no or that they did not remember. Only two women reported that they had a dream about their children a few days before giving birth and that the children in the dream looked very much like their newborns.

*M1: “Yes, a few days before the birth. I dreamt about this baby already born, I dreamt about the shape, the hair, which I later found to be the reality when she was born.”*

*M7: “Yes, yes, just before giving birth. And he was the baby I had dreamt of as soon as he was born.”*

Finally, the women interviewed talked about their emotions when holding their children for the first time. All the mothers in our sample described this moment as the happiest of their lives, and the emotions they felt as impossible to describe in words.

*M1: “An immense joy. I think it’s the best feeling a woman can have.”*

*M4: “An unspeakable emotion, absolutely, a great emotion, the greatest.”*

*M7: “An indescribable emotion, so great that you can’t even find the words.”*

*M8: “An immense, indescribable happiness, the most beautiful vision I have ever had in my life, also for my husband.”*

Only one woman added that she felt a little clumsy when she first held her daughter in her arms but that she quickly overcame this feeling with the support of her husband and family.

*M2: “A bit like clumsy because I don’t have cousins, nieces, and nephews, so I haven’t had any break-in. I haven’t had any experience. As soon as they gave me this bundle, I was alone in the hospital, but we immediately bonded. She understood, Alice my first daughter, that we were a bit inexperienced, but then we immediately got in tune, also thanks to the support of our families.”*

### 3.2. Partner, Family, and Social Support

Participants also described the reactions of family and friends to the announcement of their pregnancy. Again, most of the women interviewed reported positive responses from family and friends:

*M1: “When we announced the news, it was a moment of joy for my family, my parents, my brother.”*

*M2: “Even relatives and friends felt very emotional because they knew how much we had done to have our child.”*

*M10: “They welcomed the news with joy and enthusiasm, approval.”*

However, one of the women interviewed said that although her family and friends were all happy for her, they were at the same time worried that her child might also be born with a visual impairment:

*M2: “Everyone was happy for us. I never thought for one moment that my disability would interfere. However, as I had congenital blindness, they were a bit worried that my children might inherit the disability. But I always thought I would protect them from that, and luckily it turned out that way. But I knew that my husband had been very clear about this: if they had a problem with their sight, we would have dealt with it.”*

Only two women reported adverse reactions in their families to their pregnancies due to the belief that their disability would prevent them from taking proper care of their children:

*M3: “My mother didn’t take it well because she thought I wouldn’t be able to take care of my son because of my disability... a bit of disappointment, but otherwise everything is fine.”*

*M4: “They all took it very badly, not only our friends but also our families, but then this too was overcome…”*

In general, the women interviewed reported receiving adequate support from family and friends during pregnancy and postpartum.

*M1: “Yes, yes, fully supported. Even in the moments that followed, they were fundamental.”*

*M7: “Yes, yes. I received great support from both my family and my friends.”*

*M6: “Yes, everyone supported me. I had wonderful months of pregnancy.”*

Only one woman reported that she spent the months of her pregnancy away from her family because they lived in different cities but received support from colleagues and friends.

*M11: “During the pregnancy, I didn’t have family close by because I lived in Modena, but I had colleagues and friends who helped me.”*

Most women reported positive responses regarding the partner’s reactions to the pregnancy. In addition, the close relationship between happiness at the news and the desire to have a child emerges for the partners. Therefore, this intense desire for parenthood is a vital bonding factor within the couple.

*M2: “Also happy. Our children were born four years after we got married, so we wanted them a lot.”*

*M7: “He was also happy because we wanted it together.”*

*M12: “He was overjoyed. He wanted it as much as I did.”*

The partner did not accept the pregnancy in only one case, leaving the woman alone. In another case, the partner had some initial difficulty accepting the pregnancy due to the fear that the child would also have visual problems but overcame this resistance.

*M1: “He did not want to take responsibility, so he reacted negatively.”*

*M4: “For the first pregnancy, he had some problems with acceptance because he was afraid that our child would be born with eyesight problems, but then it went well.”*

Support from the partner was good for almost all women interviewed.

*M2: “Yes, yes, it has helped me a lot. I am fortunate.”*

*M5: “Yes, yes, very kind. I had a caring and attentive husband.”*

*M7: “Yes, he helped me, I didn’t have an easy pregnancy that forced me into bed, and he helped me.”*

The same women who reported adverse reactions of their partners to the pregnancy also said that they did not feel adequately supported.

*M1: “No, he disappeared and did not take responsibility.”*

*M4: “I didn’t feel very supported by him in the first pregnancy. For the second one, it was better.”*

When asked whether pregnancy and the birth of a child had changed their couple’s relationship, the women interviewed mainly replied that the relationship with their partner did not change or had improved due to the experience of parenthood.

*M2: “Well, of course. We used to be two people, now we are three and then four, and the family dynamics change, but I can say that our relationship has changed for the better, and we are much closer.”*

*M7: “No, our relationship has not changed at all. We have always tried to have our intimacy and not to lose it.”*

*M14: “Not so much because my husband has shown his love for me over the years.”*

Only one woman reported that her relationship with her partner changed a lot due to pregnancy and motherhood to the point of breaking up. Only one woman said that her relationship with her partner changed a lot due to pregnancy and motherhood to the point of breaking up. Another woman reported that her relationship with her partner was negatively affected during her second pregnancy by fear that the child might have health problems.

*M13: “Yes, very much so. We got to the point where our relationship turned into something bad, and we broke up.”*

*M15: “Yes, of course. Especially for the second daughter, I was afraid that something might happen to the child again, as it had happened years before. It affected my relationship, it made me feel on edge, and my reactions were not exactly appropriate.”*

Finally, concerning the social support they received during pregnancy and after the birth of their child, the women interviewed confirmed in their stories that they had to face many prejudices and preconceptions about their pregnancy and motherhood in their social network, even from family and friends. Specifically, they reported that the main misconceptions they faced were that a woman with a visual impairment could not be a mother and raise a child properly.

*M1: “They used to tell me, ‘poor girl, she won’t make it, she will need help, she won’t be able to raise her daughter alone, she will always need someone.’”*

*M2: “There is definitely a lot of preconception about disability. I think people believe that someone with a disability is not capable of being a parent.”*

*M6: “They think that we are disabled and therefore cannot give the right education to our children, but this is not the case.”*

*M9: “Many of my relatives said that I could not get married and that I could not have children. I always replied that people with disabilities also deserve to live a normal life.”*

*M12: “Because of ignorance, most people believe that people with disabilities should not have the right to love, whereas people with disabilities are more protective and responsible.”*

However, despite the prejudices and preconceptions of their social environment, all the women expressed the conviction and desire to be accepted as women and mothers by society. Furthermore, they reported that they had managed to overcome these prejudices by experiencing motherhood with joy and satisfaction.

*M8: “A lot of progress has been made compared to the taboos of the past. Many people talk openly about their disability, and I am the first to do so. The fact of asking questions is a very good thing to understand what you may need and to give answers to your doubts.”*

### 3.3. Relationship and Communication with the Child

Another central theme that emerged from the qualitative analysis of the interviews was the relationship and communication with the child.

All the women interviewed talked about how they interacted with their children and how visual impairment was an obstacle in communicating with their children. In this regard, according to the stories of the mothers in our sample, visual impairment can affect the quality of mother–child interactions, both verbal and non-verbal. In this regard, one woman reported that she experienced great difficulty and suffering from not being able to help her daughter even in small things such as “cutting her nails” and not being able to use her eyesight to communicate with her.

*M15: “It was the worst moment because I had to get very close to my daughter to see her. It was terrible; I couldn’t even cut her nails. I don’t know what our relationship was like, but I always try to do what I can. As soon as my daughter started to be self-sufficient, she started to do everything by herself, and even trivial things weighed heavily on me; the fact that I couldn’t help her even with little things like cutting her nails weighed heavily on me. With my disability, I try to do as much as possible because I don’t want to burden anyone.”*

Nevertheless, the women interviewed found alternative strategies to interact and communicate with their children, mainly based on physical contact and voice communication.

*M1: “I caressed her, songs, singing, voice, physical approach, caresses, kisses, hand cues.”*

*M2: “In all ways, with the contact having nursed, talking a lot, listening to music, the classical things mothers do.”*

*M7: “Predominantly through tactile experiences and voice, strokes, songs.”*

*M14: “I have always communicated with him. We have a very good relationship. In the first months with physical contact, I tried to interpret his sounds, to which I gave a meaning. I never allowed my son to cry hysterically because I tried to understand his discomfort through the first sounds.”*

The absence of eye contact is recognized as an obstacle in the relationship with the child by several women in our sample. More specifically, difficulties are more significant when the children are younger and tend to decrease as they grow older. The main obstacles reported during the interviews were not being present with their children at certain times, such as accompanying them to school or sports.

*M7: “The difficulty was that I could not accompany him to sports or other events while he was growing up. And I could not accompany him because of my visual impairment.”*

*M14: “It is an obstacle because it creates a form of personal discomfort that can produce displeasure in the other person. I could not always get close, and I was very sorry not to be able to distinguish his face.”*

*M15: “Yes, in some aspects. For example, I couldn’t support her when she was in primary school. She did everything by herself. She learned to read and write by herself. I try not to burden her, but I know it’s difficult.”*

When talking about how their children became aware of their mother’s disability and how they told them about their visual impairment, the women interviewed said that everything happened very naturally, and they received great support from their children in dealing with their disability.

*M1: “I started to tell it to the child when she started to speak. I said that... because obviously when she started to speak, the child asked me: “mum, why don’t you move fluidly like the other mums, why do you touch objects with your hands? And I told her because I have this disease that reduces my sight, so I need to pay more attention, to touch things, to look for them more carefully than those who have sight...”*

*M7: “When he was little, we didn’t talk about it, but as he grew up and started asking me questions, I tried to make him understand my problem.”*

*M8: “He often asked us questions that we answered naturally. We always told the truth about my and his illness. Everything was lived with clarity.”*

*M15: “When my first daughter was 11 years old, I explained to her my illness and what my problems were. She was very understanding. Both my daughters are very understanding because, for many reasons, they have grown up so quickly.”*

In particular, it is interesting to note that several women resorted to play and humor to cope with the anxiety associated with telling their children about their visual impairment.

*M2: “We even joke about my disability. You know the cartoon Mr. Magoo, have you ever seen it? It’s a cartoon from the 70s about a blind undertaker. So my husband and I joke about it; we find a funny way to justify my gaffes. The other day at the restaurant, I said: ‘How cute is that baby’, and my daughter said: ‘Mommy is a girl’. We try not to think of it as a drama.”*

*M3: “We always tried to approach everything ironically, as a game, so as not to burden our son with this situation.”*

Only one woman stated that she had never spoken openly with her child about her visual impairment and her suffering for not seeing him well.

*M14: “I have never admitted my displeasure at not being able to distinguish him. He knows that I can’t see well, but I have never said that I can’t see him perfectly.”*

### 3.4. Self-Efficacy and Self-Awareness

A final significant theme that emerged from the interviews is self-efficacy, the confidence in one’s ability to perform a task and achieve a set goal. In particular, despite the lack of confidence in their parenting skills by their social environment, the women in our sample were very confident about their ability to manage pregnancy first and motherhood later.

*M1: “I am proud of myself and the relationship I have with my daughter, how I have raised her. Despite the difficulties related to my disability, I think I am a good mother to her.”*

*M7: “I think I have been a good mother together with my husband and have raised our son with good values. That’s what counts.”*

*M14: “Yes, sometimes I am too careful. I see myself instead as a mother who lets her son free, especially now that he is grown up.”*

*M15: “I hope to be a good mother, I hope so... yes, because when I talk about it, everyone tells me that I am a good mother.”*

Nevertheless, the lack of support from the social network caused some women to have doubts and fears about their skills, which also influenced their self-efficacy. However, experiencing parenthood and becoming aware of their skills allowed these women to improve their self-efficacy concerning motherhood significantly.

*M14: “They thought I was not able to be a parent, that I had to depend on others. I was afraid of all this, but fortunately, I succeeded in my parenting. My son gives me compliments and comments on Instagram that make me very emotional.”*

These results are also confirmed by the scores of the self-efficacy scale, which the women in our sample completed during the interview with the support of the researcher. Indeed, the mean score on the questionnaire of the sample of women interviewed (*M* = 32.31; *SD* = 5.13) was not significantly different from the mean of the normative reference sample (*M* = 29.28; *SD* = 4.6). This finding, therefore, points to a good sense of self-efficacy among the women in our sample regarding their ability to cope with the tasks associated with motherhood and the difficulties related to their disability.

## 4. Discussion

This qualitative study aimed to explore, through semi-structured interviews, the experiences of a sample of visually impaired women regarding pregnancy and motherhood.

The women interviewed all experienced pregnancy and childbirth with joy and underlined their strong desire to become mothers. Furthermore, the desire for parenthood is an essential element that has helped to keep the couple together despite the difficulties. 

In particular, it emerged that it was important for these women that their desire to become mothers and their motherhood be recognized as normal by their social and family environment, as shown by other studies on women with disabilities [3]. 

However, the life stories of the women who participated in this study confirm that this desire for normality faces many prejudices about disability and especially about disabled women who wish to become mothers. More specifically, the literature on the topic underlined that women with physical disabilities are constantly confronted with the model of motherhood brought forward by today’s society based on an idea of perfect physical functioning [4,14,28]. For this reason, their parenting skills are systematically questioned, both at the family and social level [3,16]. In the specific case of the women interviewed in this study, the main prejudices related to the inability to take proper care of children were often the cause of fears and concerns about their actual parenting skills. However, as confirmed by the literature, good family and social support together with a good sense of personal self-efficacy make it possible to overcome these prejudices and experience motherhood with joy [3,7,13].

Another important theme that emerged from the qualitative analysis of the interviews concerns the relationship and communication with the child, especially during the first months of life. 

Studies on mothers with visual impairments have shown that mother–child interactions can be more difficult due to the lack of eye contact. Indeed, there is ample evidence that visual experience is fundamental to communicating emotions. In addition, emotional states are often expressed through a range of non-verbal channels such as gestures, posture, and facial expressions [29,30,31].

The non-verbal channel is critical, especially in mother–child interactions, because mothers often use visual cues such as facial expressions to detect the child’s needs or mood changes and respond appropriately [13]. Therefore, mothers with visual impairments might communicate with their children with greater difficulty than sighted mothers [32].

In this regard, the women in our sample also highlighted their difficulties communicating with their children due to the lack of eye contact. However, as confirmed by studies on the subject [13,32], these women can use alternative channels of communication, in particular physical and auditory contact. Furthermore, several studies underlined that young children of visually impaired parents could adapt more quickly to using different modes of communication to interact and communicate with their blind parents [32,33,34].

Another interesting finding concerns communication with children about disability. According to the stories of the women interviewed, communication on this issue occurred naturally, and, in several cases, play and humor played an important role in encouraging children’s acceptance of their mother’s disability. This result is in line with the literature that humor and play may have an essential coping or relief function in communicating the diagnosis of various diseases [35,36]. Furthermore, humor effectively shares personal and often sensitive information among family members [37].

The women in our sample showed a good sense of self-efficacy and high confidence in their parenting skills, which is associated with a positive experience of motherhood. This confirms the results of other studies that identify self-efficacy as an essential protective factor in mothers with visual impairment [3] because confidence in one’s abilities positively affects the power to act and significantly impacts the social environment [38,39]. 

### Strengths and Limitations

To the best of our knowledge, this study is one of the first qualitative studies on the experiences of Italian visually impaired women about pregnancy and motherhood. Furthermore, studies that have focused specifically on women with this type of disability are few and far between [12,13]. Consequently, this study represents a valuable contribution to the literature to solicit further research on the topic and appropriate support for women with visual impairments experiencing motherhood in various parts of the world. However, some limitations should be highlighted. First of all, the small sample size related to the qualitative design of the study limits the relevance of our results. Secondly, the telephone mode of interviews due to pandemic-related restrictions may bias the results. Furthermore, it is possible that the women interviewed responded in some cases following the expectations of their social and family environment. Finally, the results of our study must be read in the light of the Italian cultural and social context, so they should be cautiously generalized to the context of other countries.

## 5. Conclusions

In conclusion, mothers with visual impairments show a strong desire to be recognized and accepted as women and mothers by their social environment. However, the family and social context is often unsupportive and shows prejudice and a lack of confidence in their parenting skills. On the contrary, adequate social and family support is associated with a better sense of personal self-efficacy and greater confidence in one’s skills as a mother. 

In light of these considerations, this study highlights the need to promote an assertive and non-judgmental attitude towards women with visual impairments at both an individual and social level, especially regarding the issues of pregnancy and motherhood. Furthermore, future studies involving medical and midwifery staff regarding this topic are critical to improving health care for pregnant women and mothers with visual impairments.

## Figures and Tables

**Table 1:** Sociodemographic characteristics of the participants.

Pseudonym	Age	Disability	Number of Children	Education	Occupation	Marital Status
M1	36	Blind from birth	1	Middle school	Housewife	Live-in partner
M2	45	Blind from birth	2	Master’s degree	Housewife	Married
M3	26	Blind from birth	1	High school	Student	Live-in partner
M4	42	Blind from disease	2	High school	Housewife	Divorced
M5	56	Blind from disease	1	Primary school	Retired	Widow
M6	43	Blind from birth	1	High school	Housewife	Married
M7	49	Blind from birth	1	Middle school	Housewife	Married
M8	60	Blind from birth	1	Master’s degree	Teacher	Married
M9	56	Blind from birth	1	Middle school	Telephone operator	Married
M10	58	Blind from birth	2	Master’s degree	Teacher	Married
M11	56	Blind from disease	1	Middle school	Retired	Divorced
M12	56	Blind from birth	2	High school	Housewife	Married
M13	45	Blind from disease	2	Middle school	Housewife	Divorced
M14	52	Blind from birth	1	High school	Teacher	Married
M15	60	Blind from disease	2	High school	Housewife	Divorced

## Data Availability

The data presented in this study are available on request from the corresponding author. The data are not publicly available to ensure the privacy of the participants.
